# An Overview of the Molecular Cues and Their Intracellular Signaling Shared by Cancer and the Nervous System: From Neurotransmitters to Synaptic Proteins, Anatomy of an All-Inclusive Cooperation

**DOI:** 10.3390/ijms232314695

**Published:** 2022-11-24

**Authors:** Marco Arese, Federico Bussolino, Margherita Pergolizzi, Laura Bizzozero

**Affiliations:** 1 Department of Oncology, University of Torino, 10060 Candiolo, TO, Italy; 2 Candiolo Cancer Institute, FPO-IRCCS, 10060 Candiolo, TO, Italy

**Keywords:** tumor–nervous system molecular crosstalk, autocrine/paracrine interactions, signal transduction, hallmarks of cancer

## Abstract

We propose an overview of the molecular cues and their intracellular signaling involved in the crosstalk between cancer and the nervous system. While “cancer neuroscience” as a field is still in its infancy, the relation between cancer and the nervous system has been known for a long time, and a huge body of experimental data provides evidence that tumor–nervous system connections are widespread. They encompass different mechanisms at different tumor progression steps, are multifaceted, and display some intriguing analogies with the nervous system’s physiological processes. Overall, we can say that many of the paradigmatic “hallmarks of cancer” depicted by Weinberg and Hanahan are affected by the nervous system in a variety of manners.

## 1. Introduction

Tumors are not only composed of transformed cells; they also contain stable cell types that collectively form a complex biosystem. Endothelial cells, immunocompetent cells, cancer-associated fibroblasts (CAFs), neurons, nerves, and other elements with their products (e.g., the extracellular matrix) influence the transformed cells within the tumor microenvironment (TME) and in such a way affect tumor biology [1]. 

Targeting the TME rather the tumor cells alone has significant clinical repercussions. Indeed, drugs targeting angiogenesis, CAFs, immunocompetent cells, and some related signaling pathways are in clinical trials (reviewed in [2]). Altogether, it appears that we will better understand and tackle many open questions related to cancer only with a detailed knowledge of microenvironmental factors, their sources, and their impacts. Here, we treat the nervous system as one of these sources. 

The concept that the nervous system affects the progression of cancer was initially put forward by the Greek physician Galen, who recognized that “melancholic” women were more prone to developing breast cancer than other women (reviewed in [3,4]). The most recent indications from the burgeoning field of cancer neuroscience [5] suggest that nerves are significant players in the tumor microenvironment, engendering a selective pressure on cancer cells by modulating a complex network of mediators related to tumor progression.

There has been a considerable increase in the published research on cancer–nervous system relations in the last three years, while many excellent reviews in the last year alone [6,7,8,9,10,11] have approached the field from different perspectives. 

**Figure 1:**
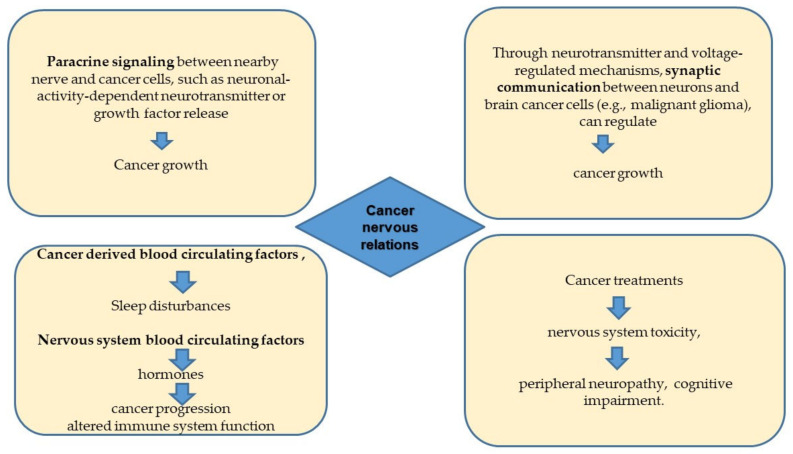
The four main areas of cancer/nervous system interactions.

Nerve and cancer cells relate in many ways, some of which are known only at the descriptive level. Taking into account the “manifest” of cancer neuroscience [5], we can cite the four main interfaces through which cancer and the nervous system influence each other: (1) Paracrine signaling between nerve cells and cancer cells, such as neuronal-activity-dependent neurotransmitter or growth factor release, regulates cancer growth in a variety of tissues. The influence of neurons on malignant cells may be mediated by other cell types in the tumor microenvironment. Paracrine factors derived from cancer remodel the nervous system to promote increased neural activity in the tumor microenvironment. (2) Synaptic communication between neurons and brain cancer cells (e.g., malignant glioma), through voltage-regulated mechanisms, can regulate cancer growth. (3) Cancer-related circulating factors can influence nervous system functions, such as sleep, whereas the nervous system can influence cancer progression via circulating factors, such as hormones and progenitor cells, or altered immune system function. (4) Cancer treatments frequently result in nervous system toxicity, ranging from peripheral neuropathy to cognitive impairment (Figure 1). 

Here, we review the molecular links between nerve and cancer cells that promote cancer progression, mainly those classified in the just-cited categories 1 and 2. Sometimes these cues have been “evolutionarily co-opted” by tumor cells from the nervous system and can have at least one function in a “tumor autonomous” manner. Obviously, even in this last category, the presence of neuron-related proteins in cancer cells strongly predicts an ability to communicate with neurons through common signaling systems. The signaling pathways of each molecular cue that we consider are briefly overviewed. Functionally, we pay particular attention to alterations in proliferative, apoptotic, survival, migratory/metastatic, or immune-regulating pathways. In more detail, our review considers: (*a*) discrete nervous system/cancer interaction sites, such as in the perineural invasion (PNI) process and the perineural niche, the sympathetic nervous system (SNS) and catecholamines, the parasympathetic nervous system (PSNS) and acetylcholine (Ach), and the tumor/nervous system interface in central nervous system tumors and electrochemical communication as well as (*b*) molecules that have widespread activity in various types of cancer and contexts: neurotransmitters/neuropeptides/amino acids/growth factors (dopamine, gamma-aminobutyric acid, serotonin, glutamate, neuropeptides, serine, and nerve growth factor (NGF)) and axon guidance/synaptic proteins. On this background, the perineural niche, with its ever-expanding role in the paracrine interactions in PNI, and the novel remarkable findings on the electrochemical interactions in brain tumors receive particular attention. 

## 2. Perineural Invasion and the Perineural Niche

Perineural invasion (PNI) is a very well known process by pathologists and one the most actively studied events in cancer–nerve relations at the molecular level. Nonetheless, our current understanding of PNI is very limited and is still highly debated. Macroscopically, it happens as cancer cells infiltrate the surrounding nerve endings, invading them through the destruction of the perineurium and entry via the perforating vessels [12].

While virtually all peripheral cancer types display PNI, the process has mostly been studied for head and neck, prostatic, and pancreatic cancers. For many of these, PNI is linked with considerable morbidity, poor outcomes, and decreased survival (reviewed in [13]).

The discrete microenvironment surrounding an invaded nerve, also called the *perineural niche*, is formed by neural cells, supporting cells, inflammatory cells, extracellular matrix, blood vessels, and immune components. The discovery of the innervated niche as a new specialized microenvironment has opened exciting new avenues of research into bidirectional neural–cancer interactions [14]. The migration of cancer cells along nerves is supported by multiple growth factors and chemokines secreted by these cell types. Nerve injury and repair, in a continuous process, seem to guide this migration (also called tracking) through the secretion of growth factors and chemokines simulating damaged and dying nerves [15]. Amit et al. [15] proposed that PNI occurs in seven stages: tumor cell survival, the formation of a neural steady state, an inflammatory reaction, the recruitment of tumor cells to nerves, the neogenesis of nerves, the adhesion of tumor cells and neurolemma, and nerve invasion. This theory is supported by data from in vitro studies, as the coculture of human prostate cancer (PCa) cells, stromal cells, and mouse dorsal root ganglia increases tumor colony and neurite outgrowth [16]. 

The molecular characterization of the perineural niche is a burgeoning field of research [17] that would require a whole review by itself. Here, we concentrate on key cellular and molecular aspects of neurogenesis and invasion, including the newly studied role of autophagy as well as new perspectives in therapy. 

Most studies emphasize the importance of neural components in tumors, making it critical to investigate the mechanism of nerve emergence in cancer. Strikingly, neurogenesis is not only a local process. Neural progenitors from the brain that express doublecortin (DCX+) can migrate to prostate tumors and initiate neurogenesis, leading to tumor growth and metastasis [18,19]. High expression of the transcription factor Snail in PCa cells improves adherence to nerve cells and promotes neurite outgrowth [19]. As a result, the neurogenesis program is crucial in prostate tumor–nerve crosstalk. A recent single-cell sequencing study discovered that Neurexin 1 and Neuroligin 1 (see below), two genes encoding neuronal synaptic molecules, are strongly linked to PCa progression [20]. Other neurotrophic factors, such as nerve growth factor (NGF) and brain-derived neurotrophic factor (BDNF), have been linked to PNI [21]. PNI in PCa is thought to be characterized by N-CAM upregulation in nerves. Li et al. proposed that cancer cells induce N-CAM upregulation in nerves via a paracrine loop that accelerates cancer cell migration towards nerves via the nuclear factor kappa B (NFkB) pathway [22]. NFkB is involved in many different types of cancer. In PCa, NFkB nuclear translocation has been found to be critical for inhibiting apoptosis and increased tumor cell proliferation in PNI lesions. Semaphorin 4F (SEMA4F), a member of the semaphorin family of axon guidance molecules (see below), is another molecule that correlates with the NFkB pathway in PNI. The SEMA4F expression level has been linked to nerve density and PNI diameter, in addition to the antiapoptotic effect induced by interacting with the NFkB pathway, as the overexpression of SEMA4F can induce neurogenesis [23]. Another semaphorin family member, semaphorin 3C (SEMA3C), has been shown to be involved in PNI in PCa. SEMA3C is activated by monoamine oxidase A (MAOA) in a Twist-1-dependent manner and interacts with its receptor and coreceptor PlexinA2 as well as neuropilin-1 (NRP1), stimulating c-MET. This SEMA3C/PlexinA2/NRP1-cMET axis promotes PNI in PCa and demonstrates the close relationship between PNI and nerve-related signals [24,25]. Many other molecular cues promoting aggressive behavior have been identified in the perineural niche. In pancreatic cancer, the cell adhesion molecule L1CAM and a metalloproteinase mediate paracrine communication between Schwann cells and cancer cells, thus promoting cancer invasion into nerves [26]. In head and neck cancer, the neuropeptide galanin (GAL) activates its receptor, GALR2, on cancer cells and stimulates the transcription of cyclooxygenase-2 and GAL itself. In turn, the cyclooxygenase-2 product prostaglandin E2 promotes cancer invasion, while the GAL released by cancer stimulates neurite outgrowth, promoting PNI [27]. In the same tumor model, the NGF-TrkA axis triggers the epithelial–mesenchymal transition (EMT) and confers resistance to the epidermal growth factor receptor tyrosine kinase (EGFR) inhibitor erlotinib [28]. Moreover, in PCa, nerve-produced glial cell line derived neurotrophic factor receptor alpha (GFRα1) activates the receptor tyrosine kinase RET, thus promoting transformation-associated phenotypes [29].

From a therapeutic perspective targeting the events at the perineural niche, two approaches are worth mentioning here. One is targeting NGF and its receptors. In prostate cancer, this may reduce nerve infiltration, inhibit tumor cell growth, and alleviate cancer-related pain (reviewed in [30]). Another possible strategy is to target the interaction of the nerve and immune cells in the tumor microenvironment. It has been reported that tumor-associated nerves in prostatic cancer have high levels of programmed cell death ligand-1 (PD-L1) expression and inhibit the function of immune cells, providing a new perspective for the use of immune checkpoint inhibitors [31].

A novel and highly investigated player in nerve cancer crosstalk is autophagy. Autophagy is a conserved cellular self-degradation process that is critical for balancing energy sources during neuronal development and nutrient stress [32,33]. Autophagy has recently been identified as a double-edged sword that plays an important role in the innervated niche [34]. The question of whether upregulated autophagy is beneficial or harmful to tumor progression in the innervated niche is still being debated. Autophagy stimulates tumor progression in part because of its ability to maintain innervated niche homeostasis and even promote innervated niche development [35,36]. In contrast, dysfunctional autophagy may result in axon breakdown, dendritic degradation, somal stress, and glial cell death, ultimately leading to innervated niche disorder [37]. As more is learned about autophagy in the innervated niche, new treatment strategies can be developed. Changing the innervated niche by regulating the autophagy pathway has emerged as a novel therapeutic opportunity for cancer treatment and a novel window for drug repurposing in this context. Following an escalating era for drug discovery, repurposing old drugs to treat new indications is becoming an increasingly appealing proposition because it is a time-saving and cost-effective method with high success rates [38,39]. A large number of preclinical trials have shown that multiple noncancer drugs (antipsychotics, cardiovascular drugs, etc.) have off-label antitumor effects [40] Based on the autophagy-mediated innervated niche, repurposing drugs such as beta-adrenergic antagonists is a promising pharmacological approach in cancer neuroscience [41,42]. Hence, it is now recognized that the innervated niche is a complex and heterogeneous site that can influence cancer development [43]. Finally, the role of a specific type of autophagy in glioblastoma progression should be cited here. Pericytes (PCs), contractile perivascular cells, are seized by glioblastoma (GB) to aid tumor progression. The work of Valdor et al. [44] showed that chaperone-mediated autophagy (CMA), a process that transports specific cytosolic proteins to lysosomes for degradation and has pro-oncogenic effects, is significantly upregulated in PCs in response to the oxidative burst that occurs after PC-GB cell interactions. The upregulation of CMA activity in PCs by GB is required for their effective interaction with GB cells, which aids tumor growth. These findings point to abnormal CMA upregulation as a mechanism by which GB cells elicit the immunosuppressive function of PCs and stabilize the GB-PC interactions required for tumor cell survival.

**Figure 2:**
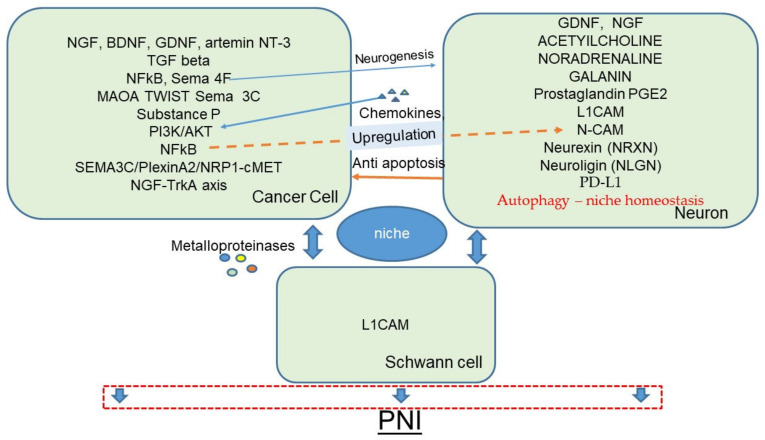
Examples of molecular mechanisms involved in PNI/perineural niche that are presented in this review. Major cell types in the perineural niche, as well as various soluble signaling molecules and their receptors, form a complex signal network that allows nerves and tumors to interact. For recent perineural-niche- and PNI-focused reviews, see [17,45].

A schematic representation of the mediators and intracellular pathways of PNI is presented in Figure 2.

## 3. The Sympathetic Nervous System and Adrenergic Signaling

The SNS regulates gene expression and cellular function throughout the body by releasing two catecholamines: norepinephrine (NE), which is secreted by SNS nerve fibers innervating target tissues, and epinephrine (E), which is a product of the adrenal gland and is released into the blood [46].

Studies of the SNS’s effects on cancer biology were originally driven by clinical observations suggesting a correlation between stress and cancer progression. Chronic stress is linked with an increased frequency of cancer [47] and is associated with poorer clinical consequences [48]. Different experimental models show that stress and increased adrenergic activity promote the progression of a range of malignancies, including prostate, pancreatic, and ovarian cancer [49,50,51,52].

Altogether, E and NE deeply affect multiple biological characteristics of cancers and their microenvironments, including resistance to apoptosis, proliferation, cell survival, invasion, metastasis, and angiogenesis [53,54,55]

E and NE signal through alpha (α)- and beta (β)-adrenergic receptors (α and β-ARs), which are G-protein-coupled receptors (GPCRs) that are broadly expressed in mammalian tissues. In particular, β2-AR are expressed on many tumor cells of epithelial and lymphoid origin. β-ARs signal through the Gαs–cyclic AMP (cAMP) stimulation of mitogen-activated protein kinases (MAPK), protein kinase A (PKA), and exchange protein activated by cAMP (EPAC). The activation of β2-ARs promotes tumor growth through vascular endothelial growth factor (VEGF) and metalloproteases, thus potentiating angiogenesis and metastasization in ovarian, lung, and breast cancer [50].

In esophageal squamous cell carcinoma, β-ARs transactivate the extracellular signal-regulated kinase (ERK)/cyclooxygenase 2 (COX2) signaling pathway, facilitating cell proliferation. In addition, the NE-β-AR axis stimulates hepatocellular carcinoma resistance to anoikis (i.e., the induction of apoptosis in cells upon the loss of adhesion) and invasion through EGFR transactivation [56]. In colon cancer, NE promotes cell proliferation and metastasis by activating the cAMP response element-binding protein-1–microRNA 373 (CREB1-miR-373) axis [57]. Recently [58], the non-receptor tyrosine kinase Src was identified as a key switch that is activated in response to beta-adrenergic signaling in cancer cells.

In pancreatic cancer, NE increases cell viability and invasion and prevents apoptosis in a Notch-1-dependent manner [59]. In the same model, catecholamines promote β2 adrenergic-dependent PDAC (pancreatic ductal adenocarcinoma) development, NGF secretion, and pancreatic nerve density. These findings suggest that catecholamines drive an upregulation of neurotrophins, which in turn increases sympathetic innervation and local NE accumulation [55]. In prostate cancer, NE reactivates the cell cycling of dormant disseminated tumor cells (DTC) through both direct actions and indirectly on adjacent cells in the bone marrow niche [60]. In a lung tumorigenesis model, NE prompts the phosphorylation of L-type voltage-dependent calcium channels (VDCC) through a β-adrenergic receptor-PKA pathway. VDCC triggers calcium mobilization, thereby causing the activation of IGF-1R via the exocytosis of insulin-like growth factor 2 (IGF2) [61].

In the prostate TME, NE inhibits oxidative phosphorylation in endothelial cells while promoting an “angiogenic switch” and cancer progression [62,63].

In an apparent contrast to the data above, exercise-induced catecholamines activate the Hippo tumor suppressor pathway in breast cancer [64]. In this context, both E and NE inhibited breast cancer cell viability as well as tumor growth in vivo. However, it is essential to distinguish between the acute and rapidly falling levels of catecholamines in response to exercise and chronic stress, which causes constant high levels of catecholamines.

The modulation of the tumor immune microenvironment by β2-adrenergic signaling is a key event in the tumor–nerve relations. Indeed, E and NE profoundly affect inflammatory immune cells in cancers [65,66,67]. For example, the activation of β-AR signaling enhances the infiltration of macrophages into the tumor parenchyma and, in so doing, induces a prometastatic gene expression signature in primary breast cancer [68]. Moreover, adrenergic signaling influences the immunosuppressive capacity of myeloid-derived suppressor cells in cancer [69]. Furthermore, the vagus nerve modulation of cytotoxic T cells in the spleen has been shown to contribute to colorectal cancer [9,70].

In melanoma, nonspecific β blockers decrease the recurrence and improve overall survival for metastatic disease treated with immunotherapy. New data show how β blockade improves cytotoxic therapies by regulating the immune response. Concurrent irradiation and beta-blockade also reduce the abundance of immunosuppressive M2-polarized macrophages and regulatory T cells, both of which have been shown to impair cytotoxic T-cell migration and the physical interaction with malignant cells in lung cancer. These findings are consistent with the widespread adrenergic and sensory innervation in lymph nodes as well as the control of T cell egress from lymph nodes via beta 2 adrenergic receptor signaling. Given that a significant portion of the immune response to immune checkpoint blockade occurs outside of the tumor site (e.g., draining lymphoid tissue), the modulation of the neural signaling that controls T-cell retention or release can have significant effects on both the native and immune checkpoint-blockade-induced immune responses [7].

## 4. The Parasympathetic Nervous System and Acetylcholine

Neurons of the PSNS typically release Ach as their primary neurotransmitter. Data show that while adrenergic signals consistently exert protumorigenic effects, cholinergic (i.e., from Ach) signals play different roles in cancer progression depending on the tumor type. Moreover, the context-dependent effects of parasympathetic signals are less understood at the molecular level, perhaps because of a paucity of tools to precisely target parasympathetic nerves [14].

Two classes of Ach receptors have been isolated: the nicotinic acetylcholine receptors (nAchRs) and the muscarinic receptors (mAchRs). nAchRs are targeted by NNK, a potent tobacco-originated carcinogen [71,72]. They signal through Ca^2+^ flows inside the cell and enhance the epithelial–mesenchymal transition, proliferation, differentiation, angiogenesis, migration, and invasion [73,74]. nAchR crosstalks with other neurotransmitter receptors, activating multiple secondary pathways, including protein kinase C (PKC), ERK1/2, COX2, prostaglandin E2 (PGE2), CREB, Src, Akt serine-threonine kinase, and the Ras-RAF1-MAPK cascade [75]. nAchRs can also prevent drug-induced apoptosis by upregulating survivin, the apoptosis suppressor gene BCL-2, and nuclear factor NF-κB in breast cancer and pleural mesothelioma.

mAchRs also modulate the progression of different cancers. Ach enhances non-small-cell lung carcinoma (NSCLC) cell proliferation through M_3_R (a subtype of mAchR)-mediated stimulation of Akt and MAPK [76]. In colon cancer [76] and gastric cancer [77,78], Ach activates M_3_R, promoting tumor progression, invasion, and metastasis. In contrast, PDAC tumors respond differently to cholinergic stimulation. In these tumors, a muscarinic agonist inhibits pancreatic cancer progression by inhibiting MAPK and phosphoinositide 3-kinase (PI3K)/Akt signaling [79]. Accordingly, resection of the vagus nerve (which carries parasympathetic and sensory inputs) accelerates cancer progression [79,80]. In breast cancer, cholinergic nerves also display an inhibitory role: in one instance, constitutive increases in cholinergic activity by the exogenous expression of long-acting bacterial sodium channels were found to prevent cancer growth [81].

Ach displays an anti-inflammatory activity, and the vagus nerve has a unique role in inhibiting proinflammatory cytokine production [82,83]. Most inflammatory cells express mAChRs and nAChRs and respond to autocrine and paracrine stimuli [82].

One final comment is that, while less diffuse than its adrenergic counterpart, cholinergic signaling also has a role in the neuroregulation of the tumor immune microenvironment. In a mouse model of pancreatic cancer, muscarinic receptor activation lowers TNFα levels in the blood and the spleen, indicating that cholinergic signaling generates an anticancer immune microenvironment [79]. In pancreatic cancer, PNI is associated with both acetylcholine levels and reduced CD8+ T and Th1 cell abundance, while a bilateral vagotomy in mice increased CD8+ T cells in the tumors and resulted in longer survival [7,84].

## 5. The Tumor/Nervous Interface in the Central Nervous System Tumors and Electrochemical Communication

As could probably be anticipated from all the evidence outlined above, tumor–nervous system interactions can also be observed in primary and metastatic tumors of the central nervous system, where neurons account for approximately half of all cells. Interestingly, two recent publications demonstrated that gliomas (brain tumors derived from glial cells) grow by forming excitatory synapses with neurons in the brain [85,86]. Even more suggestively, cancer cells totally unrelated to the nervous system in origin, i.e., breast cancer metastases, form excitatory synapses with glutamatergic neurons in the brain [87]. These are very remarkable discoveries because they put the arguably most peculiar and functionally advanced neuronal structure, the synapse, in a shared context with tumor cells, further highlighting the cleverness of evolution, which uses its best “molecular solutions” in all possible settings, thus saving energy.

The issue of electrical neuronal input in facilitating cancer proliferation, particularly in brain tumors, deserves further analysis. Glioma cells are affected by electrical activity in the same way as the normal human nervous system. Gliomas, in turn, release growth stimuli into the surrounding tissue, including glutamate, increasing neuronal activity in the microenvironment. Given the presence of calcium permeable AMPA-receptor dependent synapses in high-grade gliomas, glioma-derived glutamate contributes to a bidirectional positive feedback loop of tumor–nerve hyperactivity and tumor proliferation [86].

The electrical integration of brain tumors with neuronal networks causes some clinical manifestations of gliomas, such as seizures, to be reconsidered. Seizures can directly result from glutamate secreting tumors, augmenting neural hyperactivity, despite being commonly regarded as a byproduct of the tumor’s mass effect or surrounding edema. Indeed, glioma subtypes with higher synaptogenic potential have been linked to increased seizure frequency and tumor invasion [88], and recurrent postoperative seizures have been linked to glioblastoma tumor recurrence. [89] These findings raise the question of whether antiepileptic drugs can be used to treat cancer. Preliminary research has looked into various antiepileptic drugs in glioblastoma, but more research is needed. Much remains unknown about how electrical activity from the nervous system influences cancer growth. Although the precise roles of electrical input are still unknown, the current understanding of electrical networking is consistent with the idea that tumors are driven not only by cell intrinsic processes but also by the intricately connected components of their surrounding microenvironment (reviewed in [7]).

## 6. Molecules with a Widespread Activity in Various Types of Cancer: Neurotransmitters, Amino Acids, Growth Factors, Axon Guidance, and Synaptic Proteins

### 6.1. Dopamine

Dopamine is a catecholamine family member and a precursor of NE and E. It regulates several key functions from cardiovascular activity to behavior. In peripheral tissues, dopamine is produced by sympathetic neurons, neuroendocrine cells, and the adrenal medulla (reviewed in [90]). Dopamine binds to five different G-protein-coupled receptors (DRs).

In general, there is mixed evidence regarding the influence of dopamine on cancer, suggesting that its role might be dependent on the tumor type, the expressed receptors, and the experimental conditions.

Dopamine or DR agonists inhibit the growth of gastric cancer, breast cancer, and sarcoma [91]. However, in glioblastoma, the inhibition of a DR subtype inhibits the proliferation and survival of cancer stem cells [92]. In pancreatic cancer, the inhibition of another DR subtype reduces cell migration and proliferation and decelerates the growth of experimental tumors in mice [93]. In yet another tumor model, no effect was detected on the proliferation and invasion of colon cancer cells upon DR modulation [94].

A more consolidated mechanism of the tumor-suppressive effect of dopamine may be associated with decreased angiogenesis [95]. Interestingly, dopamine prevents the release of endothelial progenitor cells from the bone marrow by blocking VEGFA-mediated ERK1/2 phosphorylation [96].

Finally, it is plausible that the antitumor effects of dopamine occur by the modulation of various immune-competent cells within the TME [97]. Dopamine also inhibits the function of a subtype of myeloid-derived suppressor cells through D1-like receptors [98] and regulates the activity of peritoneal macrophages and regulatory T lymphocytes [99].

### 6.2. Gamma-Aminobutyric Acid

Gamma-aminobutyric acid (GABA) is the main inhibitory neurotransmitter in the central nervous system. GABA activity in cancer is multifaceted and appears to be heavily context-dependent. Importantly, the GABA cell content is augmented in several types of human tumors, including glioma, gastric cancer, and breast cancer [100,101,102].

GABA binds two classes of cellular receptors: (*a*) the ionotropic chloride channels GABA_A_ and GABA_C_ receptors, and (*b*) the metabotropic GABA_B_ receptors, which are related to the catecholaminergic receptors.

Many tumor tissues express GABA receptors [103,104,105], and with a few exceptions, the GABA_A_ receptor mediates the GABA-induced proliferation of cancer cells, while the GABA_B_ receptor mediates the GABA-induced inhibition of cancer cell growth [105].

The GABA_A_ receptor is upregulated in breast, pancreatic, and prostate cancer [106,107,108]. In pancreatic cancer, GABA_A_ activation increases intracellular Ca^2+^ levels and stimulates the MAPK/ERK cascade and tumor growth [109]. Muscimol, a GABA_A_ receptor agonist, increases the proliferation of gastric cancer cells by activating the MAPK cascade.

The GABA_B_ receptor is expressed at low levels in pancreatic and liver cancer [110,111]. GABA or GABA_B_ agonists decrease stomach carcinogenesis in rats [112], inhibit experimental metastasis formation in colon cancer [113,114], and decrease cell growth in human hepatocellular carcinoma both in vitro and in vivo [110]. The activation of GABA_B_ receptors strongly inhibits adrenergic-induced ERK1/2 phosphorylation and prevents DNA synthesis and cell migration [115]. On the other hand, GABA itself or baclofen (a GABA_B_ agonist) promote the invasion of prostate cancer cells by the transactivation of EGFR [116,117].

Altogether, a complex connection between GABA and cancer seems to exist, with the differential activities probably mediated by the cancer-specific expression and signaling of its receptors.

Importantly, GABA is present in the TME, raising the likelihood that it might target the infiltrated immune cells [107].

Globally, the GABAergic system may directly or indirectly modulate cancer biology and therefore be a potential therapeutic target. Drug repurposing is within reach, as GABA is broadly used in therapy, for example, as an antidiabetic agent [118].

### 6.3. Serotonin

Serotonin (5-hydroxytryptamine or 5-HT) is a monoamine neurotransmitter with a wide array of functions, from cognitive and behavioral functions to various peripheral roles in the intestine, blood vessels, and immune system [119].

5-HT is a potent mitogen for many types of nontumoral cells [120,121] as well as for tumor cells from the lung, pancreas, colon, bladder, and prostate [122,123,124,125,126]. However, the serotonin-induced signaling pathways in cancer are complex and only partially understood (reviewed in [127]).

The wide activities of 5-HT are mediated by 5-HT receptors (5-HT_1–7_) that are extensively distributed in the human body. Two subtypes of receptors, 5-HT_1A_ and 5-HT_1B_, are overexpressed in high-grade prostate cancer cells [128], while antagonists of these receptors induce apoptosis [123,124].

Epithelial tumors often display abnormal 5-HT signaling. Indeed, 5-HT regulates epithelial homeostasis in the mammary gland, lung, pancreas, and prostate [127,129]. In particular, a series of analogues of the potent 5-HT1B/1D serotonin receptor agonist 5-nonyloxytryptamine (5-NT) induce apoptosis and interfere with signaling pathways that regulate protein translation and survival, such as the Akt/mTOR pathway, in triple-negative breast cancer cells [130]. Human pancreatic cancer tissues contain high amounts of 5-HT and its receptor, HTR2B. Under these circumstances, tumor glycolysis under metabolic stress is allowed and tumor growth is promoted [131].

Finally, platelet-released 5-HT stimulates tumor growth, angiogenesis, and metastatic spread [132]. Remarkably these activities of 5-HT appear to be mediated by endothelial nitric oxide synthase and p-ERK1/2 [133].

Globally, while data suggest that 5-HT signaling is involved in cancer biology, further studies are necessary to clarify which contexts will benefit from a 5-HT-targeted therapy.

### 6.4. Glutamate

The amino acid glutamate is a fundamental excitatory neurotransmitter in the mammalian CNS that is involved in a wide array of functions from learning to affective behavior. Glutamate is also involved in the central biosynthetic and bioenergetics pathways.

Abnormal glutamate signaling contributes to tumorigenesis in a variety of cancers [134,135], including colorectal [136], glioma [137], gastric [138], oral squamous cell carcinoma [139], and melanoma [140].

Glutamate binds two classes of receptors: ionotropic and metabotropic. Three classes of ionotropic glutamate receptors are known: AMPA, NMDA, and kainate receptors. Conversely, metabotropic receptors (mGluR) are GPCRs constituting a large family containing several subgroups.

NMDA receptor expression has a prognostic value in oral squamous cell carcinoma; in particular, the overexpression of NMDAR1 correlates with the TN stage, the size of the primary tumor, and the metastatic spread [141].

mGluR is also involved in cancer. In colon cancer, high expression of mGluR4 is related to poor prognosis [142], while the activation of mGluR4 protects cells from 5-fluorouracil (5-FU) toxicity; conversely, in the same model, reduced mGluR4 expression or its drug-induced inactivation lead to cell death [136]. In medulloblastoma [143], however, mGluR4 expression is inversely correlated with cancer aggressiveness. The glutamate-mGluR axis can lead to the phosphorylation of the p110β subunit of phosphoinositide 3-kinase, facilitating prostate cancer progression [144]. Thus, the clinical implication of glutamate receptor expression may vary among the various tumor types.

While in general the data accumulated so far suggest the opportunity for the development of therapeutic agents targeting glutamate signaling, more studies are requested regarding the function of glutamate in the immune TME, a key target of cancer therapy.

### 6.5. Serine

A very recent publication showed that the amino acid serine, secreted by sensory and sympathetic neurons, stimulates pancreatic cancer metabolism in nutrient-lacking conditions [145]. These notable findings widen the array of cellular phenotypes affected by tumor–nervous system connections and put serine on the list of small molecules of neuronal origin that exert a selective pressure on cancer cells.

### 6.6. Neuropeptides

Neuropeptides are peptides that neurons use to communicate with the environment. Different neuropeptides modulate a wide range of brain activities but can also function peripherally as endocrine/paracrine factors regulating various physiological processes [146].

Substance P (SP) [147] and neuropeptide Y (NPY) [148], with their receptors of the GPCR superfamily, are the neuropeptides that have been studied the most in cancers. SP modulates the apoptosis, proliferation, migration, angiogenesis, and invasion of many cancers, including colon, pancreatic, melanoma, osteosarcoma, gastric, glioma, larynx, and lung carcinoma [147,149]. The biological activity of SP is primarily mediated by the neurokinin-1 (NK-1) receptor and two second messengers: diacylglicerol (DAG) and inositol 1,4,5-triphosphate (IP3) [149]. Interestingly, the inhibition of the NK-1 receptor with specific pharmacological antagonists results in pronounced antitumor effects [150]. Other specific roles of SP/NK1 in cancer can be described. In breast cancer, the miRNA MiR-34b/c-5p and its target NK-1 receptor affect cell proliferation and apoptosis. SP rescues the effects of miR-34b/c-5p overexpression or NK1 receptor silencing on cell proliferation and apoptosis in in vitro and in vivo assays [151]. In glioma, SP, through the NK-1 receptor, controls cancer cell proliferation by activating c-myc, MAPK, activator protein 1, and ERK 1/2. In glioma cells, SP promotes glycogen degradation, which is essential for glycolysis. By contrast, antagonists of the NK-1 receptor stop the proliferation of tumor cells and the degradation of glycogen and stimulate apoptosis [152].

NPY is a growth-enhancing factor in numerous cancers, including Ewing sarcoma, neuroblastoma, and breast and prostate cancer [148,153]. NPY modulates not only tumor growth and vascularization but also the metastatic and chemoresistant phenotypes of cancer cells [154,155]. NPY, which is abundantly produced by enteric neurons, also regulates carcinogenesis via two systems: first by the downregulation of microRNA-375-dependent apoptosis in intestinal epithelial cells and second by enhanced proliferation (via PI3-K/pAkt) [156].

While the mechanisms through which neuropeptides regulate tumor progression is only partially known, the existing evidence supports the possibility of therapeutically targeting neuropeptides and their receptors.

### 6.7. Nerve Growth Factor

A nervous growth factor that deserves particular mention is NGF because of its widespread activity in cancer biology. Solid cancers respond to NGF of autocrine/paracrine origin. TrkA and p75NTR are both receptors for NGF that are dysregulated in breast cancer, prostate cancer, and melanoma. The binding of these receptors results in downstream PI3K pathway, MAPK pathway, and c-Jun N-terminal kinase (JNK) pathway activation, which drives increased tumor survival, proliferation, and invasion [157].

Many solid tumors, including gastric cancer, also release NGF in an effort to influence the surrounding neurons and the TME [78]. In breast cancer, the levels of NGF correlate with both the density of tumor innervation and tumor aggression. This supports the hypothesis that tumor-released NGF stimulates neuron growth and the innervation of the tumor. The newly formed axons, in turn, release tumor growth factors, thereby driving tumor aggression. Remarkably, NGF-induced neurons often express tyrosine hydroxylase, further supporting the existence of a positive pressure of sympathetic adrenergic signaling on cancer evolution through increased tumor aggressivity and treatment resistance [158].

### 6.8. Axon Guidance Molecules and Neuroligin

In this paragraph we initially cover the role in cancer of three main “classic” families of axon guidance molecules (netrins, ephrins, and semaphorins). Many excellent reviews have covered these molecules in the context of cancer [159,160,161,162], so we mainly outline the latest findings. Finally, we report on our newer findings on the tumor autonomous role of the synaptic protein neuroligin.

#### 6.8.1. Axon Guidance Molecules

*Netrins* are soluble proteins that act through their receptors of the DCC (deleted in colorectal cancer) or UNC5 families and determine the direction of neuronal neurites [163]. Netrins have, however, been found to play a role in tumor biology [160,164], where in particular DCC and UNC5 positively regulate apoptosis in the absence of netrin-1 but negatively regulate apoptosis in the presence of netrin-1 [165]. Reduced netrin-1 expression is present in tumors of the prostate and the nervous system [166,167]. In intestinal cancer, DCC suppresses tumor growth by inducing cancer cell death when netrin-1 expression is low [168]. However, netrin-1 and its receptor DCC also control the progression of melanoma, indicating that the therapeutic targeting of this signaling axis may be a viable method for melanoma treatment [169]. Interestingly, it has also been shown that netrin-1 is elevated in cancer-associated fibroblasts (CAFs) in addition to cancer cells [170]. In addition to its direct impact on cancer cells, netrin-1 inhibition may hence inhibit the crosstalk between pro-neoplastic CAFs and cancer cells, reducing malignant plasticity.

Mechanistically, it was discovered that a portion of colorectal cancers exhibit a netrin-1 increase concurrent with transcription factor NF-kB activation and that netrin-1 is a direct transcriptional target of NF-kB [171]. Additionally, colorectal malignancies in the context of inflammatory bowel disorders (IBD) exhibit an overexpression of netrin-1. Numerous data point to a close connection between chronic inflammation and tumorigenesis, primarily via NF-kB activation. Hence, it was suggested [171] that an NF-kB-mediated increase in netrin-1 expression in IBD patients may have an impact on the promotion and progression of colorectal tumors. Moreover, netrin-1 upregulates YAP (an oncoprotein located in the cytoplasm in an inactive form that, when activated, translocates to the nucleus and activates the transcription of genes responsible for cell division and apoptosis) levels in the nucleus, while inactivating netrin-1, DCC, or UNC-5B decreases YAP protein levels [172].

The Eph receptors, with their ligands the Ephrins, are a large family of transmembrane proteins that were originally implicated in many neurological functions [173,174]. Eph/ephrin signaling is exceptionally complicated because of its bidirectional nature (with Ephs and ephrins interacting “in trans” from two different cells), and as a consequence Eph/ephrin have been shown to coordinate a plethora of tumor-related processes from angiogenesis, inflammation, vascular infiltration, and metastasis to tumor immunity [175,176,177]. To name a few examples, increased Eph/ephrin signaling is associated with tumor progression in melanoma [178], breast [179], prostate [180], and colorectal cancer [175]. The Eph family member EPHA2 has recently been proposed as a negative prognostic factor in triple-negative breast cancer (TNBC) patients; moreover, EPHA2 may act as a precision therapeutic target for this challenging condition [181]. Finally, EphB2 signaling is increased in Barrett’s esophagus and esophageal adenocarcinoma and regulates the MYC oncogene [182]

Mechanistically, after an EPH-ephrin engagement, a complex molecular pathway activates signaling in both cells that are involved. EPHs and ephrins engage with different molecular cascades to further transmit the message into the cytoplasm. Forward signaling involves interactions between EPHs and Src family kinases, which regulate the development of synapses; Rho GTPases, which stabilize junctions; and interactions between ephexins (GEFs that can activate Rho GTPases) and the ERK/MAPK pathway, which promote cell proliferation. Additionally, FAK and the JAK/STAT pathway interact with EPHs, which modifies cell adhesion. The interactions between ephrins and Src, Erk, Rac, paxillin, and p75, which result in integrin-dependent cell adhesion, as well as with Src, Grb4, PTP-BL, and PDZ-RGS3, which control a variety of actions, including cell adhesion, migration, and proliferation, are among the mechanisms implicated in reverse signaling. Phosphoinositide 3-kinase is also known as PI3K Janus kinase, JAK RGS3 stands for regulator of G-protein signaling 3, FAK stands for focal adhesion kinase, and STAT stands for signal transducer and activator of transcription [175].

*Semaphorins* are a large family of secreted, transmembrane, or GPI-anchored proteins (SEMAs) signaling through their receptors, neuropilins, and plexins. As for many other cues considered here, semaphorins were originally described as guidance molecules for neurons but were then found to be released by several types of cancer cells [183,184] and by cells in the tumor microenvironment [185]. Semaphorins impact many cancer hallmarks: cancer cell proliferation, angiogenesis, immune response, invasion, and metastasis [186].

Regarding the inflammatory and immunological roles of semaphorins, their receptors are abundantly expressed in inflammatory cells, and tumor cells produce their ligands, resulting in intense local and systemic signaling crosstalk. Additionally, many semaphorins regulate immune cells that are both innate and antigen-specific. Notably, semaphorin signals that function as inhibitors of the immune system’s ability to fight cancer are frequently dysregulated in human malignancies and could potentially be targeted for treatment [187].

To illustrate the complexities of the biological activities and signaling of the semaphorin family members in cancer with an example, we take the case of breast cancer, based on the excellent work of Butti and colleagues [188]. Sema3A binds to the neuropilin 1 (NRP1) receptor, ultimately inhibiting tumor growth and angiogenesis. Sema3B binds to NRP1 and inhibits PI3K/Akt signaling, causing apoptosis. Full-length Sema3C interacts with NRP2 on lymphatic endothelial cells in tumors, inhibiting VEGF-C-dependent ERK1/2 and Akt signaling and suppressing lymphangiogenesis and metastasis. Sema4D binds to plexin-B1 and activates ErbB2, phosphorylating plexin-B1. By activating RhoA GTPase, phosphorylated plexin-B1 promotes migration. Through ErbB2-dependent MAPK signaling, cleaved p61-Sema3E binds to plexin-D1 to promote metastasis. Sema3E binds to plexin-D1 and disrupts the interaction between plexin-D1 and NR4A, which is known to induce caspase-9-mediated apoptosis [188].

Overall, notwithstanding their complicated signaling mechanism, research in the field of semaphorins is active in order to develop anticancer strategies [186,189].

#### 6.8.2. Neuroligins

Synaptic proteins involved in cancer include postsynaptic scaffold proteins, receptors, and ion channels and have been very recently reviewed [6]. Moreover, it is particularly worth noting the extensive expression of components of the synaptic machinery, such as synaptophysin, in some non-neural cancers [190]. This is easily noticeable, for example, in small-cell lung carcinoma, a very aggressive type of lung cancer, which displays many similarities with neuronal cells, and some forms of colon and prostate cancers [190]. This attractive biological phenomenon could reflect the selection of cancer cells endowed with the molecular tools needed to interact with the nervous system.

Here, we only consider a novel synaptic protein that we and others have discovered to be involved in cancer: neuroligin 1.

The family of neuroligins is composed of transmembrane postsynaptic proteins of the CNS that function in the fine-tuning of synaptic activity. We have previously shown that a member of this family, neuroligin 1 (NLGN1), is expressed in endothelial cells and modulates angiogenesis [191,192,193,194]. While a database study by Qian et al. [195] showed that NLGN1 is expressed in a subset of colon tumors and has negative prognostic implications, very recently we demonstrated that NLGN1 is expressed in human colorectal tumors, in particular in groups of aggressively migrating cells); single tumor cells from primary tumors (an event called tumor budding), which represents a negative prognostic factor; and in circulating tumor cells as either single cells or aggregates (that are referred to as ‘emboli’ or ‘microemboli’). We discovered that NLGN1 enhances CRC cell extravasation/lung invasion, differential organ metastasization in two mice models, and CRC cell transendothelial migration across an endothelial monolayer in vitro. In CRC cell lines, NLGN1 upregulates mesenchymal markers and WNT target genes, enhances APC (adenomatous polyposis coli, a CRC tumor suppressor that is mutated in up to 80% of CRC samples) localization to the cell membrane, co-immunoprecipitates with some isoforms of this protein, and induces beta-catenin translocation to the nucleus. NLGN1 appears as the first eminently synaptic neuronal protein that modulates CRC aggressiveness by affecting a crucial pathogenetic route of this disease [196].

Before this report, we demonstrated that NLGN1 is expressed by prostatic and pancreatic cancer tissues in distinct stages and tumor districts using immunohistochemistry on human tissue microarrays. Then, using in vitro and in vivo tests, we were able to show that NLGN1 stimulates cancer cells to invade and move along nerves. We investigated a potential cooperation between NLGN1 and the neurotrophic factor glial cell line derived neurotrophic factor (GDNF) because this factor is known to play a function in tumor–nerve interactions. We discovered that the NLGN1-induced in vitro cancer cell invasion of nerves was fully prevented by suppressing GDNF activity with a specific antibody. Finally, we showed that GDNF significantly activates cofilin, a cytoskeletal regulating protein, in the presence of NLGN1, changing filopodia dynamics. Here, it was demonstrated that NLGN1 interacts with one of the most prevalent neurotrophic factors in the nervous system, potentially opening new treatment avenues [197].

Another neuroligin isoform (NLGN3) has been involved in glioma growth. This protein is structurally and functionally related to NLGN1 (72% aa identity and 81% conservation), as they are both present at excitatory synapses. The protease ADAM10 (a disintegrin and Metalloprotease 10) is stimulated by neuronal activity to promote NLGN3 extracellular domain shedding into the tumor microenvironment, which promotes glioma growth [198]. A clinical trial (#NCT04295759, https://clinicaltrials.gov/ (accessed on 17 October 2022)) is underway to test the efficacy of the medication INCB7839 in inhibiting ADAM10 in gliomas. It is important to note that in the brain ADAM-10 similarly sheds the NLGN1 ectodomain in an activity-dependent manner [199]. Therefore, future studies may look at whether soluble NLGN1 can function as a mediator of CRC growth and metastasization.

## 7. Conclusions

**Figure 3:**
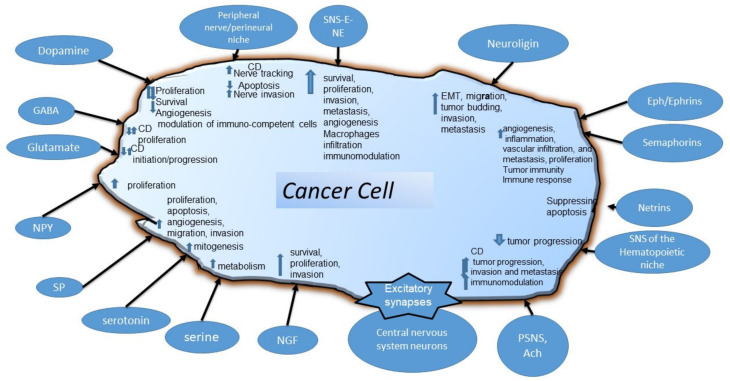
Outline of the tumor cell/ nervous system interfaces and their biological effects on cancer cells that have been cited in this review. Up arrows indicate the upregulation of a certain pathway/phenotype. Down arrows indicate downregulation. CD: context-dependent, means that variable responses depend on the tumor type and various other experimental conditions. Intra- and intertumor heterogeneity most likely play a role. All other abbreviations can be found in the main text.

A number of very recent reviews have comprehensively analyzed tumor/nervous system crosstalk from different perspectives [7,14,200,201]. Here, we aimed to comprehensively analyze the shared molecules and signaling pathways between the systems and their biological outcomes. Figure 3 and Table 1 present a quick overview of the molecules that we considered, their signaling, and their biological effects.

Our review has left out some molecular cues (e.g., some members of the neurotrophins and the neuropoietin family) with very specific activities that are presented in other more dedicated publications [202,203,204].

We have collectively shown that many of the original hallmarks of cancer [205] are affected by its interaction with the nervous system. We believe that these include acquired capabilities for sustaining proliferative signaling, resisting cell death, inducing/accessing the vasculature, activating invasion and metastasis, reprogramming cellular metabolism, and avoiding immune destruction. Hence, our review certainly sustains the idea that the molecular tumor–nerve crosstalk may represent a further hallmark of cancer on its own, as recently proposed [10].

As was certainly noticed, except for the case of the adrenergic input on epithelial tumor growth, the effects of the nervous system mediators on cancer biology are extremely multifaceted and complex. In the case of the differential effects of the SNS and PSNS, one can make an interesting parallel with the complementary physiological effects that these two branches of the autonomic nervous system have in the human body.

In general, the effect of a given neural cue is heavily dependent on the considered isoform and the tumor model used. Although at times this may be due to different experimental settings, we believe that this situation mostly reflects the extensive innate inter- and intratumor heterogeneity, both among the transformed cells and their microenvironments. In particular, different regions of tumors display different molecular compositions of the extracellular matrix, different quantities of infiltrating normal cells, and different amounts of blood and lymphatic vessels. In this setting, single tumor cells within a mass are subject to a range of microenvironmental inputs and respond with a variety of phenotypic manifestations. We believe that, as for other microenvironmental cues, the nervous system is heavily involved in this diversity.

Finally, it should be remembered that the goal of innervation is not only to transmit rapid signals between nerves and tissues. Denervation and cross-reinnervation experiments in striated muscle have shown that neuronal signals regulate the phenotypic traits of innervated target tissues [206,207]. This is a key observation for the study of nervous influence on tumors. In fact, if this is also the case for nerve–tumor cell interactions, whereby a selective force is imposed by the nervous system on the phenotypic features of cancer cells, then the stimulation/inhibition of nerves entering tumors, the denervation of tumors, or the modulation of the molecules reviewed here might offer new therapeutic options.

## Figures and Tables

**Table 1:** A schematic presentation of the neurotransmitters/amino acids/growth factors considered in this review, with molecular data. More details on the intracellular pathways, including the abbreviations used, can be found in the main text and references.

Family	Member	Cellular Receptor	Affected Intracellular Pathways	Refs
Neurotransmitters/amino acids	Norepinephrine (noradrenaline)	α-β adrenergic receptors	Gαs–cAMP, MAPK, PKA, EPAC ERK/cyclooxygenase 2 CREB1-miR-373 axis, Src PKA, VDCC, Hippo	[50,56,58,61,64]
	Dopamine	Dopamine 1–5 (D1–5) receptors	VEGFA-mediated ERK1/2 phosphorylation	[95,96]
	Serotonin (5-hydroxytryptamine)	5-HT1-7 receptors	Akt/Mtor, glycolysis, endothelial nitric oxide synthase, p-ERK1/2	[130,131,133]
	Acetylcholine	Muscarinic acetylcholine receptor, nicotinic acetylcholine receptor	Ca2+ flux, PKC/ERK1/2, COX2, PGE2, CREB, SRC, AKT, Ras-RAF1, MAPK, survivin, BCL-2, NF-κB, PI3K/AKT	[2,5,73,75,76,79]
	Glutamate	Metabotropic glutamate receptor, NMDA receptor, Kainate receptor, AMPA receptor	PI3K (p110)	[144]
	Gamma-aminobutyric acid	GABA_B_ GABA_A_ GABA_c_ receptors	Ca2+ flux MAPK/ERK	[103,109]
	Serine	Amino acid transporters	mRNA translation	[145]
Neuropeptides	Neuropeptide Y	NPY1R, NPY2R, PPYR1, NPY5R	PI3-K/pAkt microRNA-375	[156]
	Substance P	NK1, NNK2, NNK3	inositol 1,4,5-triphosphate (IP3) DAG, c-myc, mitogen-activated protein kinases, activator protein 1, extracellular signal-regulated kinases 1 and 2, glycogen breakdown	[149,152]
Growth factors	NGF	TrkA, p75NTR	PI3K, MAPK c-Jun N-terminal kinase	[157]
Axon guidance	Netrin 1	DCC, UNC5	Netrin 1 is a target of NFkB and induces YAP.	[171,172]
	Ephrins	Ephs	Forward signaling: Src family kinases, Rho GTPases, Ephexins (GEFs that can activate Rho GTPases), ERK/MAPK pathway, which promote cell proliferation. Additionally, FAK and the JAK/STAT pathway interact with EPHs, which modifies cell adhesion. Reverse signaling: interactions between ephrins and Src, Erk, Rac, T, paxillin, p75, and integrin-dependent cell adhesion as well as with Src, Grb4, PTP-BL, and PDZ-RGS3, which controls a variety of actions, including cell adhesion, migration, and proliferation.	[175]
	Semaphorins	Neuropilins, plexins	Sema3A: nrp1 PTEN/FOXO 3a-dependent MelCAM (CD146) expression. Sema3B binds to NRP1 and inhibits PI3K/Akt signaling. Full-length Sema3C NRP2 inhibits VEGF-C-dependent ERK1/2 and Akt signaling and suppresses lymphangiogenesis and metastasis. Cleaved Sema3C promotes cancer cell survival. Sema4D binds to plexin-B1 and activates ErbB2, phosphorylating plexin-B1. By activating RhoA GTPase, phosphorylated plexin-B1 promotes migration. Through ErbB2-dependent MAPK signaling, cleaved p61-Sema3E binds to plexin-D1 to promote metastasis. Sema3E binds to plexin-D1 and disrupts the interactions between plexin-D1 and NR4A, which is known to induce caspase-9-mediated apoptosis.	[188]
Synaptic proteins	Neuroligin 1	-	Neuroligin 1 recruits APC to the plasma membrane, blocking beta catenin degradation and causing its transfer to the nucleus, where it promotes EMT-linked gene transcription.	[196]
	Neuroligin 1	-	Neuroligin 1 synergizes with GDNF to induce cancer cell invasion of nerves and to activate the cytoskeleton-regulating protein cofilin.	[199]
	Neuroligin 3	-	NLGN3 extracellular domain shedding by ADAM10 into the tumor microenvironment promotes glioma growth.	[198]
	For all other synaptic proteins, see [6].			[6]

## Data Availability

Not applicable.
